# The Performance of Satellite-Based Links for Measurement-Device-Independent Quantum Key Distribution

**DOI:** 10.3390/e23081010

**Published:** 2021-08-03

**Authors:** Guoqi Huang, Qin Dong, Wei Cui, Rongzhen Jiao

**Affiliations:** 1 School of Science, Beijing University of Posts and Telecommunications, Beijing 100876, China; huangguoqi@bupt.edu.cn (G.H.); dongqin@bupt.edu.cn (Q.D.); cuiwei@bupt.edu.cn (W.C.); 2 State Key Laboratory of Information Photonics and Optical Communication, Beijing University of Posts and Telecommunications, Beijing 100876, China

**Keywords:** measurement-device-independent quantum key distribution, satellite-based links, quantum communication network

## Abstract

Measurement-device-independent quantum key distribution (MDI-QKD) protocol has high practical value. Satellite-based links are useful to build long-distance quantum communication network. The model of satellite-based links for MDI-QKD was proposed but it lacks practicality. This work further analyzes the performance of it. First, MDI-QKD and satellite-based links model are introduced. Then considering the operation of the satellite the performance of their combination is studied under different weather conditions. The results may provide important references for combination of optical-fiber-based links on the ground and satellite-based links in space, which is helpful for large-scale quantum communication network.

## 1. Introduction

Quantum key distribution (QKD) can generate keys to encrypt information. Theoretically, based on quantum mechanics, the security is absolutely guaranteed [1,2]. In 1984, BB84 protocol, the first QKD protocol, was proposed by Bennett [3]. After BB84 protocol was proposed, decades have passed. QKD has much progress [4]. However, due to the imperfect technology, there are some unavoidable security leaks [5,6,7]. Methods for different leaks are raised [8,9,10]. In 2012, MDI-QKD protocol was put forward by Lo [11], which can solve the problem of unsafe measurement, and it has been improved [12,13]. It adds an untrust measurement Charlie for bell-state measurement. Besides, Alice and Bob generate keys according to the postelection. In practice we use a weak coherent pulse (WCP) [14] source to emit single photon, whose number of photons obeys Poisson Distribution. So it must have multiphoton part. For this part, there is an attack called photon-number splitting (PNS). In order to solve this problem, decoy-state protocol was proposed and combined with MDI-QKD [15,16,17]. In this protocol, Alice and Bob can generate different intensities’ pulses to hide the real signal states so that no one knows whether it is signal state except senders. Now MDI-QKD protocol with decoy-state is promising. It can communicate in optical-fiber-based links, and the max communication distance is up to 404 km [12], which can be widely used.

However, the optical-fiber-based links’ loss limits the max communication distance and it leads to difficulties of building the long-distance quantum communication network. Quantum repeater, relying on quantum memory, is a way to solve this problem. We can connect many short-distance links to achieve long-distance communication. In 2021, Li and Zhou et al. [18] reported an elementary link of a quantum repeater based on absorptive quantum memories, which is a promising way of conducting quantum repeater’s scheme. Another way is using satellite-based links to realize quantum key distribution in long distance. Classical light in atmosphere has been well improved [19,20,21]. Quantum light in atmosphere has also made some progress [22,23,24,25]. From 2016 to 2020, Vasylyev et al. [26,27] showed model of probability distribution of transmittance (PDT) in atmosphere under different weather conditions; Liorni et al. [28] applied PDT to satellite-based links and studied BB84 protocol in satellite-based links; Liang et al. [29] used MDI-QKD protocol instead of BB84 protocol in [29]. In 2021, Pan’s team [30] reported an integrated space-to-ground quantum communication network by BB84. They used Micius satellite to connect quantum communication network on the ground over 4600 km. However, Liorni et al.’s work [28] using BB84 is not safe enough and satellite’s position and weather conditions are single in Liang et al.’s work [29], which lead to lack of practicality. Considering that the operation of the satellite and different weather conditions, this paper further researches on the performance of satellite-based links for MDI-QKD protocol when the satellite’s height is from 500 km to 2000 km and the angle from zenith is from $0^{\circ }$ to $80^{\circ }$.

## 2. Theory

**Figure 1:**
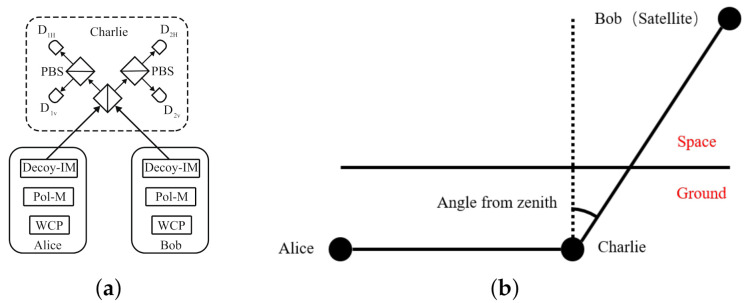
(**a**) Schematic of MDI–QKD. (**b**) Schematic of satellite-based MDI–QKD.

MDI-QKD protocol in optical-fiber-based links model is shown in Figure 1a. The classical MDI-QKD with polarization state consists of Alice, Bob and Charlie. Alice and Bob prepare polarization states and encode them. Then Alice and Bob send them to untrust Charlie for the Bell Measurement. Alice and Bob use the Decoy-IM to add decoy-state. Considering the above, we can get the key rate of MDI-QKD,

(1) $$R\geq P_{11}^{Z}Y_{11}^{Z}\left[1-H_{2}\left(e_{11}^{X}\right)\right]-Q_{\mu_{a}\mu_{b}}^{Z}f_{e}H_{2}\left(E_{\mu_{a}\mu_{b}}^{Z}\right),$$

where X(Z) is the diagonal(rectilinear) basis; $Q_{\mu_{a}\mu_{b}}^{Z}$ and $E_{\mu_{a}\mu_{b}}^{Z}$ are the gain and quantum bit error rate; $Y_{11}^{Z}$ and $e_{11}^{X}$ are the single-photon yield in the *Z* basis and the single-photon error rate; $\mu_{a}$($\mu_{b}$) is Alice(Bob)’s signal intensity; $P_{11}^{Z}$ is the probability that both Alice and Bob send single-photon; $f_{e}$ is the error correction inefficiency function; $H_{2}$ is the binary entropy function given by $H\left(x\right)=-x\log_{2}\left(x\right)-\left(1-x\right)\log_{2}\left(1-x\right)$.

We need practical parameters in Table 1 to get key rate. $e_{d}$ is the total misalignment error, $e_{0}$ is the error probability of vacuum pulses, $P_{d}$ is the dark count of each detector, $f_{e}$ is the error correction inefficiency, *a* is the loss of fibers, $\eta_{det}$ is the detector efficiency.

MDI-QKD protocol with satellite-based links model is shown in Figure 1b with main parameters on it. There are optical-fiber-based links between Alice and Charlie, which is on the ground, and satellite-based links between Bob and Charlie, which is in the air and space.

For MDI-QKD protocol, we care about the transmittance of fibers. When applying it to Figure 1b, we should consider the same. In Liorni et al.’s work [28], the transmittance is affected by lot of factors but many of them have fixed distribution. Hence, we only get PDT. Here, we consider the operation of the satellite (the height and the angle from zenith) and weather, such as light intensity, turbulence, scattering particles and so on. Weather conditions’ simulation relies on parameters of Table 2. $C_{n}^{2}$ is the value of the refractive index structure constant, $n_{0}$ is the density of scattering particles.

After PDT is gotten from [10], the average key rate of MDI-QKD with satellite-based links can also be gotten:

(2) $$\overset{-}{R}=\int_{0}^{1}R\left(\eta \right)P\left(\eta \right)d\eta =\sum_{i=1}^{N_{bins}}R\left(\eta_{i}\right)P\left(\eta_{i}\right)$$

where $R\left(\eta \right)$ is the average key rate as a function of transmittance, which can be gotten by Equation (1); $P\left(\eta \right)$ is the PDT; $N_{bins}$ is the quantity of PDT sampling.

## 3. Calculation Results

For MDI-QKD protocol, we chose vacuum + weak decoy states in the same scheme as [11]. For satellite-based links, it includes Down-link and Up-link and PDT is different through different links. However, both of them have the same simulation method. In this paper we only show Down-link.

**Figure 2:**
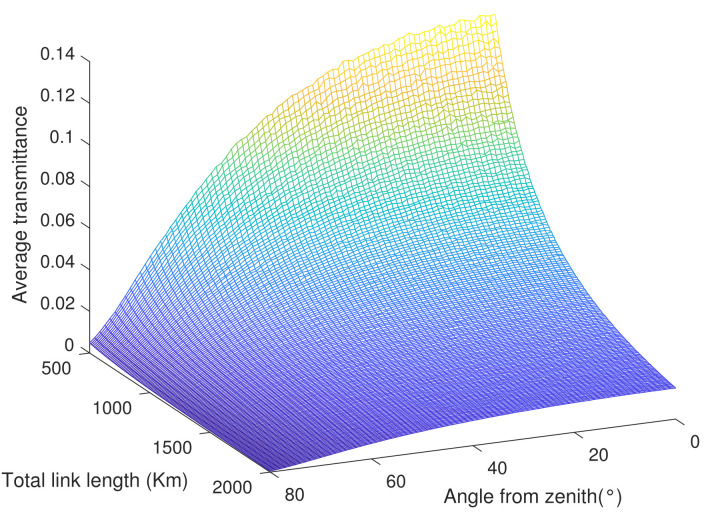
The convexity of average transmittance with the orbit’s height and the angle from zenith changing in condition 1.

By using PDT and Equation (2), Figure 2 is gotten. It only cares about the relationship between the average transmittance and the operation of the satellite visually, which is useful to the following researches. From Figure 2, the average transmittance is up to the max when the satellite is closed and nearly vertical to the ground and decreases with increasing height and angle.

**Figure 3:**
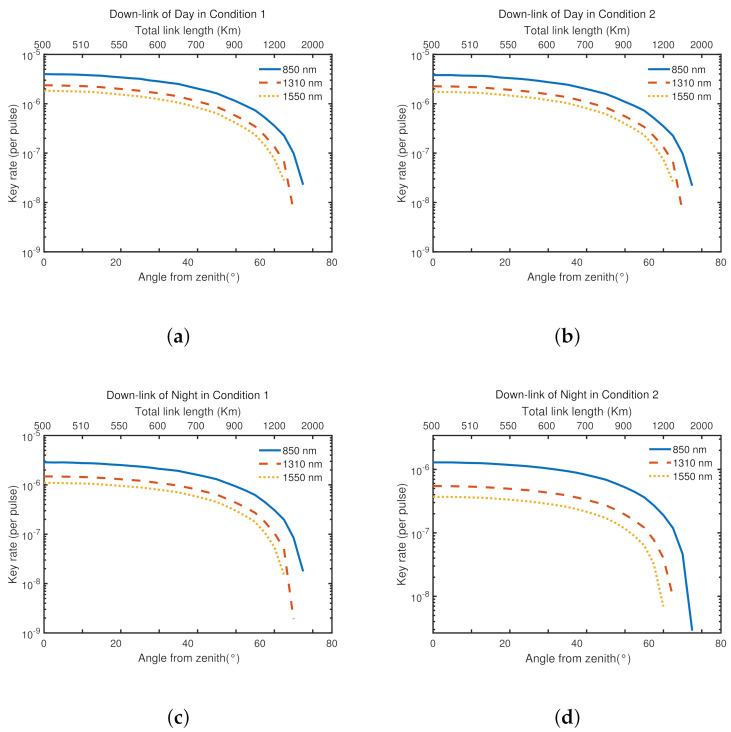
The secret key rates of the MDI-QKD protocol in two different weather conditions, as functions of the angle from zenith and the orbit’s height, are reported at signal light’s wavelength λ=850 nm, 1310 nm, 1550 nm respectively. $L_{A}$ is fixed at 50 km.

**Figure 4:**
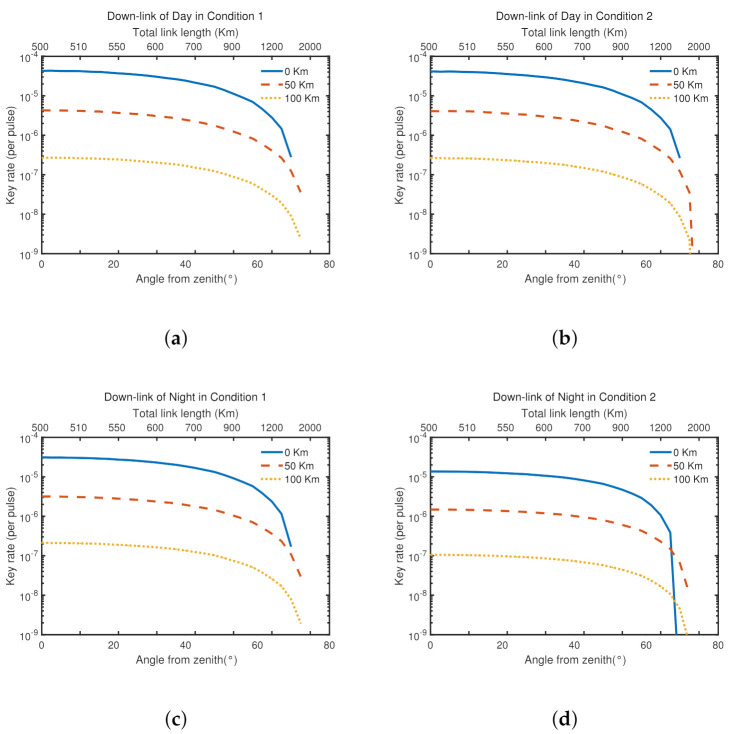
The secret key rates of the MDI-QKD protocol in two different weather conditions, as functions of the angle from zenith and the orbit’s height, are reported at $L_{A}$ = 0 km, 50 km, 100 km respectively. λ is fixed at 785 nm.

Considering that the height of satellite and the angle from zenith and the weather conditions, we simulate and get Figure 3 and Figure 4. λ is the signal light’s wavelength. It helps to study general optical communication window in satellite-based links. $L_{A}$ is the length of optical-fiber-based links and also the distance between Alice and Bob on the ground.

As we can see, by the longitudinal comparison, due to the weather such as the light and so on, the average key rate is higher in the same condition during the day than that at night.; by the horizontal comparison, in different weather conditions, it changes little during the day, but changes a lot at night.

Figure 3 shows that the large tolerance of general optical communication window. Changing the wavelength of signal light has little effect on the average key rate. In Figure 4, we can find that the ground loss has large effect on the average key rate. When $L_{A}$ = 100 km, the lowest average key rate is almost as low as $10^{-9}$. Besides, when the satellite operates between (500 km, 0${}^{\circ }$) and (700 km, 45${}^{\circ }$), the average key rate is relatively stable. The average key rate decreases fast when the satellite’s height and angle from zenith exceed (700 km, 45${}^{\circ }$). It almost reaches 0 at (1500 km, 75${}^{\circ }$), which means that it can hardly communicate with MDI-QKD when the satellite is too high and almost parallels to the ground.

From the two figures, what we should be noticed is the large effect of the loss of optical-fiber-based links on the ground. However, it also can cover a general city. The large tolerance of general optical communication window helps combine optical-fiber-based links with satellite-based links.

## 4. Conclusions

In this paper, the performance of satellite-based links for measurement-device-independent quantum key distribution has been further evaluated. The effect of weather conditions, the different wavelengths of signal light and the ground loss on the model is analyzed. All the results are reported by changing the satellite’s height and the angle from zenith. Compared with previous work, it is more close to the reality. It combines the traditional optical-fiber-based links with free-space links. It is significant for long-distance quantum communication. Moreover, it also offers important reference to build long-distance quantum communication network with satellite-to-ground links. More progress can be made in transmittance and key rate and better results may be gotten with optimizing by machine learning.

## Figures and Tables

**Table 1:** List of practical parameters of MDI-QKD for numerical simulations [11].

$e_{d}$	$e_{0}$	$P_{d}$	$f_{e}$	*a*	$\eta_{\det}$
1.5%	0.5	$3\times 10^{-6}$	1.16	0.2	14.5%

**Table 2:** List of practical parameters of weather conditions [10].

Night	Condition 1	Condition 2
$C_{n}^{2}$	$1.12\times 10^{-6}$ m${}^{-2/3}$	$5.50\times 10^{-6}$ m${}^{-2/3}$
$n_{0}$	0.61 m${}^{3}$	3.00 m${}^{3}$
**Day**	**Condition 1**	**Condition 2**
$C_{n}^{2}$	$1.64\times 10^{-6}$ m${}^{-2/3}$	$8.00\times 10^{-6}$ m${}^{-2/3}$
$n_{0}$	0.01 m${}^{3}$	0.05 m${}^{3}$

## Data Availability

Data sharing not applicable.

## Abbreviations

The following abbreviations are used in this manuscript:

QKD	Quantum key distribution
MDI-QKD	Measurement-device-independent quantum key distribution
WCP	Weak coherent pulse
PDT	Probability of transmittance

## References

[1] Ekert A.K. (1991). Quantum cryptography based on Bell’s theorem. Phys. Rev. Lett..

[2] Gisin N., Ribordy G., Tittel W., Zbinden H. (2001). Quantum cryptography. Rev. Mod. Phys..

[3] Bennett C.H., Brassard G. (2014). Quantum cryptography: Public key distribution and coin tossing. Theor. Comput. Sci..

[4] Pirandola S., Andersen U.L., Banchi L., Berta M., Bunandar D., Colbeck R., Englund D., Gehring T., Lupo C., Ottaviani C. (2020). Advances in Quantum Cryptography. Adv. Opt. Photonics.

[5] Huttner B., Imoto N., Gisin N., Mor T. (1995). Quantum Cryptography with Coherent States. Phys. Rev. A.

[6] Scarani V., Bechmann-Pasquinucci H., Cerf N.-J., Dušek M., Lütkenhaus N., Peev M. (2009). The security of practical quantum key distribution. Rev. Mod. Phys..

[7] Lo H.K., Curty M., Tamaki K. (2015). Secure quantum key distribution. Nat. Photonics.

[8] Hwang W.-Y. (2003). Quantum Key Distribution with High Loss: Toward Global Secure Communication. Phys. Rev. Lett..

[9] Lo H.K., Ma X., Chen K. (2005). Decoy State Quantum Key Distribution. Phys. Rev. Lett..

[10] Wang X.B. (2005). Beating the Photon-Number-Splitting Attack in Practical Quantum Cryptography. Phys. Rev. Lett..

[11] Lo H.K., Curty M., Qi B. (2012). Measurement-device-independent quantum key distribution. Phys. Rev. Lett..

[12] Yin H.L., Chen T.Y., Yu Z.W., Liu H., You L.X., Zhou Y.H., Chen S.J., Mao Y., Huang M.Q., Zhang W.J. (2016). Measurement-Device-Independent Quantum Key Distribution Over a 404 km Optical Fiber. Phys. Rev. Lett..

[13] Dellantonio L., Sørensen A.S., Bacco D. (2018). High dimensional measurement device independent quantum key distribution on two dimensional subspaces. Phys. Rev. A.

[14] Agnesi C., Lio B.D., Cozzolino D., Cardi L., Bakir B.B., Hassan K., Frera A.D., Ruggeri A., Giudice A., Vallone G. (2019). Hong-Ou-Mandel interference between independent III–V on silicon waveguide integrated lasers. Opt. Lett..

[15] Zhou Y.H., Yu Z.W., Wang X.B. (2015). Making the decoy-state measurement-device-independent quantum key distribution practically useful. Phys. Rev. A.

[16] Wang Q., Zhou X.Y., Guo G.C. (2016). Realizing the measure-device-independent quantum-key-distribution with passive heralded-single photon sources. Sci. Rep..

[17] Jiang C., Yu Z.W., Wang X.B. (2016). Measurement-device-independent quantum key distribution with source state errors in photon number space. Phys. Rev. A.

[18] Liu X., Jun H., Li J.F., Li X., Li P.Y., Liang P.J., Zhou Z.Q., Li C.F., Guo G.C. (2021). Heralded entanglement distribution between two absorptive quantum memories. Nature.

[19] Tatarskii V.I. (1971). The Effects of the Turbulent Atmosphere on Wave Propagation.

[20] Ishimaru A. (1978). Wave Propagation and Scattering in Random Media.

[21] Andrews L.C., Philips R.L., Hopen C.Y. (2001). Laser Beam Scintillation With Applications.

[22] Diament P., Teich M.C. (1970). Photodetection of Low-Level Radiation through the Turbulent Atmosphere. Opt. Soc..

[23] Vasylyev D.Y., Semenov A.A., Vogel W. (2012). Toward Global Quantum Communication: Beam Wandering Preserves Nonclassicality. Phys. Rev. Lett..

[24] Sidhu J.S., Joshi S.K., Gundogan M., Brougham T., Lowndes D., Mazzarella L., Krutzik M., Mohapatra S., Dequal D., Vallone G. (2021). Advances in Space Quantum Communications. arXiv.

[25] Semenov A.A., Vogel W. (2010). Entanglement transfer through the turbulent atmosphere. Phys. Rev. A.

[26] Vasylyev D., Semenov A.A., Vogel W. (2016). Atmospheric Quantum Channels with Weak and Strong Turbulence. Phys. Rev. Lett..

[27] Vasylyev D., Semenov A.A., Vogel W., Günthner K., Thurn A., Bayraktar Ö., Marquardt C. (2017). Free-space quantum links under diverse weather conditions. Phys. Rev. A.

[28] Liorni C., Kampermann H., Bruß D. (2019). Satellite-based links for quantum key distribution: Beam effects and weather dependence. New J. Phys..

[29] Liang W., Jiao R.Z. (2020). Satellite-based measurement-device-independent quantum key distribution. New J. Phys..

[30] Chen Y.A., Zhang Q., Chen T.Y., Cai W.Q., Liao S.K., Zhang J., Chen K., Yin J., Ren J.G., Chen Z. (2021). An integrated space-to-ground quantum communication network over 4,600 kilometres. Nature.

